# Optimizing Embryo Collection for Application of CRISPR/Cas9 System and Generation of Fukutin Knockout Rat Using This Method

**DOI:** 10.3390/cimb46050234

**Published:** 2024-04-23

**Authors:** Dong-Won Seol, Byoung-Jin Park, Deog-Bon Koo, Ji-Su Kim, Yong-Hyun Jeon, Jae-Eon Lee, Joon-Suk Park, Hoon Jang, Gabbine Wee

**Affiliations:** 1 Preclinical Research Center, Daegu-Gyeongbuk Medical Innovation Foundation (KMEDIHUB), Daegu 41061, Republic of Korea; seol@kmedihub.re.kr (D.-W.S.); jeon9014@kmedihub.re.kr (Y.-H.J.); koof12@kmedihub.re.kr (J.-E.L.); jsp@kmedihub.re.kr (J.-S.P.); 2 Non-Clinical Evaluation Center, Osong Medical Innovation Foundation (KBIO Health), Cheongju 28160, Republic of Korea; 3 Primate Resources Center, Korea Research Institute of Bioscience and Biotechnology (KRIBB), Jeongeup 56212, Republic of Korea; parkbj@kribb.re.kr (B.-J.P.); kimjs@kribb.re.kr (J.-S.K.); 4 Department of Biotechnology, Daegu University, Gyeongsan 38453, Republic of Korea; dbkoo@daegu.ac.kr; 5 Department of Life Science, Jeonbuk National University, Jeonju 54896, Republic of Korea

**Keywords:** Fukuyama congenital muscular dystrophy, *fukutin* gene, knockout rat model, CRISPR/Cas9

## Abstract

Rat animal models are widely used owing to their relatively superior cognitive abilities and higher similarity compared with mouse models to human physiological characteristics. However, their use is limited because of difficulties in establishing embryonic stem cells and performing genetic modifications, and insufficient embryological research. In this study, we established optimal superovulation and fertilized–egg transfer conditions, including optimal hormone injection concentration (≥150 IU/kg of PMSG and hCG) and culture medium (mR1ECM), to obtain high-quality zygotes and establish in vitro fertilization conditions for rats. Next, sgRNA with optimal targeting activity was selected by performing PCR analysis and the T7E1 assay, and the CRISPR/Cas9 system was used to construct a rat model for muscular dystrophy by inducing a deficiency in the *fukutin* gene without any off-target effect detected. The production of *fukutin* knockout rats was phenotypically confirmed by observing a drop-in body weight to one-third of that of the control group. In summary, we succeeded in constructing the first muscular dystrophy disease rat model using the CRISPR/CAS9 system for increasing future prospects of producing various animal disease models and encouraging disease research using rats.

## 1. Introduction

Transgenic animals generated in the laboratory through the artificial insertion or deletion of genes of interest in their genomes provide immense academic and economic value when expressing new phenotypes. Transgenic animals are most commonly used to model human diseases, and rodents such as mice and rats are most commonly used for this purpose because they are easy to raise and handle and possess excellent reproductive abilities [1]. Between rats and mice, rats hold the practical advantage because they are genetically and physiologically more similar to humans [2,3]. Nevertheless, the number of genetically engineered rat models is limited despite these advantages. As rat embryonic stem (ES) cells are more difficult to culture than mouse ES cells, the production efficiency of ES cell-based transgenic rats is low [3,4]. Hence, mice animal models are currently preferred owing to the abundant pool of transgenic mice. The recent and rapid development of genome editing technologies such as the clustered and regularly interspaced short palindromic repeat (CRISPR) system has led to a pressing need to establish knockout (KO) systems using rats.

Muscular dystrophy (MD) is a group of hereditary heterogeneous muscle diseases that are characterized by the inhibition of glycosylation of alpha-dystroglycan (α-DG) in muscle fibers, which results in reduced binding activity with laminin, the degeneration and weakness of the skeletal muscle, and final progression to severe physical disability and various complications [5]. Fukuyama congenital muscular dystrophy (FCMD) is the most common type of MD in Japan, with FCMD gene mutation occurring in approximately 80% of the Japanese population [6,7]. The FCMD gene encodes Fukutin, and FCMD is a genetic disease that is caused by an autosomal recessive mutation in the 10-exon of the FCMD gene, leading to muscle weakness, hypotonia, mental retardation, and meningitis in infancy [6,8,9,10]. A function-deficient mouse model that has been established helped reveal that fukutin is essential for embryonic survival [11] in addition to its vital role in the development of the nervous system [12], heart [13], and skeletal muscle [14,15]. However, the fukutin-deficiency model has only been established in mice.

In this study, we generated a KO rat model using the CRISPR/Cas9 system to investigate the functional role of fukutin in MD. First, we identified the optimal hormone injection concentration and culture medium to obtain high-quality zygotes that did not affect development in a rat model. Second, we selected the target region for inducing deficiency in the rat fukutin gene and confirmed the success and efficiency of establishing knockout using the CRISPR/Cas9 system. Next, we successfully produced two function-deficient rats by microinjecting the CRISPR/Cas9 system into the pronuclei of rat-fertilized eggs. We confirmed that the rats that were produced displayed a phenotype with 40% less weight and skeletal muscles than that of the control group at 7 weeks of age. We anticipate that the function-deficient rats thus established would be valuable for elucidating the mechanism underlying MD and its progression, and aid in developing subsequent treatment strategies.

## 2. Materials and Methods

### 2.1. Animal Experiments

All animal experiments used 9–12-week-old female Sprague–Dawley (SD) rats purchased from Koatech (Pyeongtaek, Gyeonggi-do, Republic of Korea). They were housed in the SPF facility of the Laboratory Animal Center of Daegu-Gyeongbuk Medical Innovation Foundation (DGMIF) under a 12:12 h light:dark cycle at a temperature of 23 ± 1 °C and humidity of 60 ± 5%. The rats were allowed ad libitum access to a standard rodent diet and water. All experiments were approved by the DGMIF Institutional Animal Care and Use Committee (IACUC) (license number: KMEDI-23070602-01) and were performed in accordance with the institutional guidelines for the care and use of laboratory animals. To obtain ovulated eggs for pronuclear microinjection, 10-week-old female SD rats were intraperitoneally injected with pregnant mare serum gonadotropin (PMSG; Dsmbio, Seoul, Republic of Korea) at the following concentrations: 75, 150, and 250 IU/kg. After 48 h, human chorionic gonadotropin (hCG; Sigma-Aldrich, St. Louis, MO, USA) was injected intraperitoneally at the same concentrations. The animals were mated with 10-week-old males. The next morning, female rats with confirmed vaginal plugs were euthanized and the oviducts were collected. Next, the zygote was recovered by perfusing the oviduct with M2 medium (Sigma-Aldrich), followed by washing thrice and culturing in an M16 medium (Sigma-Aldrich) drop under mineral oil in an incubator at 37 °C and 5% CO_2_. The next day, the rat embryos (that were at the 2-cell stage) were transferred to M16 medium and modified rat 1-cell embryo culture medium (mR1ECM; COSMO BIO, Carlsbad, CA, USA) to observe early embryonic development.

### 2.2. Generation of sgRNA and Selection of Optimal Target

Using the Rgen Tool program (Korea Institute for Basic Science; http://www.rgenome.net/about/; 6 April 2023), we analyzed the DNA sequence of the fukutin gene in the rat genome and selected the following three target sequences: 5′-CCTCGGCTGGATGGAATAGACTC-3′, 5′-CCTCTGCACTATGTCTGTAAGCT-3′, and 5′-CCTCACTCCAGATTTATCGAGTC-3′. To confirm the off-target effect, the DNA base sequence of the target region was confirmed using the Cas-Offinder program of Rgen Tool. The sgRNA and Cas9 systems used the px330 with the U6 and CMV promoters. A preliminary study was performed to select the system with the optimal efficiency among the constructed CRISPR/Cas9 systems. Fibroblasts were derived from rat ear tissue. The isolated fibroblasts were cultured at 37 °C and 5% CO_2_ using DMEM (Welgene, Gyeongsan, Gyeongsangbuk-do, Republic of Korea) culture medium containing 10% FBS (Welgene), 1% MEM NEAA (Gibco, Waltham, MA, USA), and 1% Penicillin Streptomycin (Gibco). Next, transfection with the sgRNA-Cas9 expression vector was performed using Lipofectamine 3000 (Sigma-Aldrich), and the genomic DNA was extracted after 2 days. Finally, the sgRNA with optimal targeting activity was selected by performing PCR analysis and T7E1 assay.

### 2.3. PCR Analysis and T7E1 Assay

Genomic DNA samples were prepared from ear fibroblasts to identify knockout efficiency. Tail biopsies were performed to identify founder rats with potential targeted mutations in fukutin. PCR was performed using Emerald Amp GT PCR Master Mix (2× premix, Takara, Shiga, Japan) according to the manufacturer’s manual. The following primers were used: for Target 1 and 2—forward, 5′-CCCATTCTGCACAGCTTTC-3′, reverse, 5′-GACACTCAGCACTATAGGAC-3′; and for Target 3—forward, 5′-GTCTGGTGTTCAAGATCCTG-3′, reverse, 5′-GCATCATCTTCAACTCTGCC-3′. Agarose gel electrophoresis-based genotyping was performed to confirm product size. Next, to conduct a mismatch detection assay, the PCR products were denatured at 95 °C for 20 min; re-annealing was performed at a −0.5 °C/s ramp to 85 °C, followed by a −1 °C/s ramp to 25 °C. The hetero-complexed product (20 µL) was incubated with 10 U T7E1 enzyme (New England Bio Labs, Ipswich, MA, USA) at 37 °C for 1 h. The assay products were electrophoresed on a 2% TBE agarose gel (Takara). The samples for which gene targeting was confirmed using the T7E1 assay were TA-cloned, and DNA sequencing (Bioneer, Deajeon, Republic of Korea) was performed to confirm the indel (insertion/deletion) pattern.

### 2.4. DNA Microinjection into Fertilized Eggs

Thirty hours after mating, the oviducts were isolated from female rats, and fertilized eggs were collected by flushing. As the collected zygotes were mixed with cumulus cells, the cumulus cells were removed using M2 culture medium containing 0.1% hyaluronidase. Next, fukutin sgRNA and Cas9 protein (Toolgen, Seoul, Republic of Korea) were mixed at a 1:2 ratio and incubated for 10 min at 37 °C for activation. The activated sgRNA and Cas9 protein were injected into the male pronucleus of the fertilized eggs using a microinjector and observed to detect the swelling of the pronucleus. Then, the zygotes were washed with M16 culture medium and incubated at 37 °C with 5% CO_2_ for 1 h to select viable fertilized eggs, followed by fertilized egg transfer.

### 2.5. Hormone Injection and Embryo Transfer into Surrogate Mother

To optimize the hormone dose, female SD rats were injected with 75, 150, or 250 IU/kg of PMSG; the same amount of hCG was administered 48 h later. Depending on the experimental group, hormone-injected SD rats were mated with normal male SD rats overnight, and the vaginal plug was checked the following morning.

To induce pseudopregnancy, female SD rats were mated with vasectomized SD rats overnight and vaginal plugs were checked the following morning. Selected transgenic fertilized eggs were surgically transferred into the oviduct ampulla of surrogate mother rats. Embryo transfer was performed in three surrogate rats, and each received 30 embryos. In total, four independent experiments were performed. The surrogate mothers were monitored after embryo transfer, and the number of pups born was recorded. To confirm the presence or absence of gene targeting in the baby rats, gDNA was isolated from the tail tissue and subjected to PCR and genotyping using T7E1 and TA cloning.

### 2.6. Analysis of Skeletal Muscle Fiber

Skeletal muscle tissue (quadriceps) was fixed in 10% formaldehyde at room temperature for four days. For hematoxylin and eosin staining, the sections were stained for 2 min in hematoxylin, 1 min in eosin, and then dehydrated with ethanol and xylene. The slides were washed with 0.5% glacial acetic acid, followed by dehydration and mounting. Images were captured using an ECLIPSE Ni-L microscope (Nikon, Tokyo, Japan). ImageJ software v1.54 (NIH, Bethesda, MD, USA) was used for analysis.

### 2.7. Statistical Analysis

The data are presented as means ± standard error of the mean (SE). The results of all experiments were analyzed using a one-way analysis of variance (ANOVA) or *t*-test following Bonferroni’s Multiple Comparison Test. All data were analyzed using the GraphPad Prism software 5.0 (San Diego, CA, USA). The statistically significant differences in the results are expressed as *p* < 0.05.

## 3. Results

### 3.1. Establishment of Optimal Condition of Embryo Experimental System in Rat Animal Model

**Figure 1:**
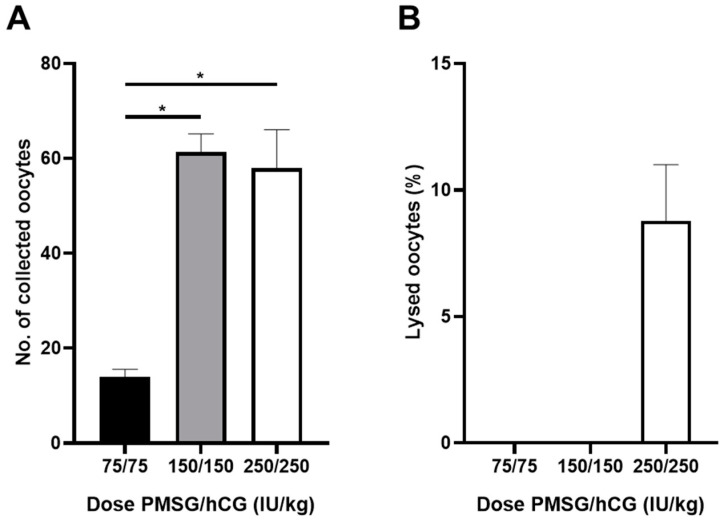
Establishment of optimal superovulation of experimental embryonic system in rat animal model. (**A**) Comparison of superovulation rates according to doses of PMSG and hCG. (**B**) Comparison of dead zygotes according to doses of PMSG and hCG. *Asterisks indicate significant differences, * *p* < 0.05.

After performing the procedure to determine the optimal hormone concentrations for superovulation (as described in the methods), the zygotes were collected from each group of mice, counted, and analyzed for survival. On average, 60 zygotes were collected per rat in the 150 and 250 IU/kg injection groups, respectively; however, only 14 zygotes were collected per rat in the 75 IU/kg group (Figure 1A). No zygote death was observed in the 75 and 150 IU/kg treatment groups; however, approximately 10% death was confirmed in the 250 IU/kg treatment group (Figure 1B).

Next, to examine whether hormone concentration and culture medium affected embryo development, the in vitro development of each group of zygotes was analyzed at various hormone concentrations and with different media. We found that, at the two- to four-cell stage, the development in the 150 IU/kg M16 medium group was significantly increased compared with that of the other groups; however, in the mR1ECM medium group, no significant difference was observed based on hormone dose. At the 8–16 cell and blastocyst stages, development was not observed among the M16 medium groups, whereas among the mR1ECM medium groups, significantly higher development was observed related to the 150 IU/kg hormone dose compared to other doses (Table 1). Taken together, these results suggest that the optimal hormone concentration for superovulation and embryo development in rats is 150 IU/kg.

Additionally, to determine whether the zygotes were implanted through embryo transfer development into normal pups, the zygotes from rats superovulated using 150 IU/kg hormone dose were implanted four times into the oviduct at the rate of 30 embryos per surrogate mother rat. After 3 weeks, all surrogate mothers (100%) gave birth and raised offspring normally. On average, the numbers of pups born were 9.3 in the first experiment, 6 in the second experiment, 7.7 in the third experiment, and 9 in the fourth experiment (Figure S1). Taken together, these results confirm the establishment of a stable superovulation and transgenic embryo transfer system.

### 3.2. Construction of CRISPR/Cas9-Based Rat Fukutin Gene Knockout System

**Figure 2:**
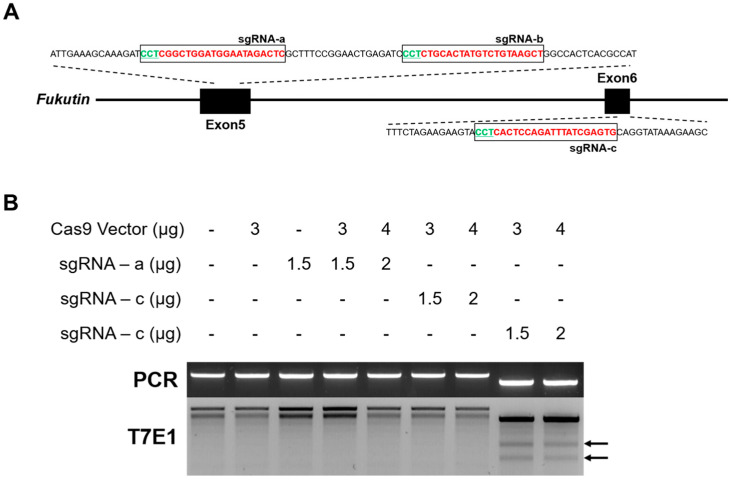
Construction of CRISPR/Cas9-based rat fukutin gene knockout system. (**A**) Target sequences recommended by CRISPR RGEN tools in rat fukutin gene exon. The target region is represented in red text, and the PAM sequence is represented in green text. sgRNA sequences are indicated in order as a, b, and c. (**B**) Verification of efficiency of designed sgRNA. The three recommended types of sgRNA and CRISPR/Cas9 vector were each transfected into rat fibroblasts, and 2 days later, gDNA was isolated and PCR and T7E1 analyses were performed. The arrow indicates that the gene targeting was successful.

To produce a rat model of MD, a rat fukutin gene knockout system was constructed using the Rgen Tool program. Among the 10 fukutin exons, three target sites were selected by targeting the first half of the exon, and selecting exons 5 and 6 with high probability of frameshift mutations in the amino acid sequence and a low likelihood of off-target mutations (Figure 2A, a–c). The first and second targets are located in exon 5 with an out-of-frame score of 77.6% and 73%, respectively, as well as no mismatch in the rat genome, and the third candidate is located in exon 6 with an out-of-frame score of 84.1%, and no mismatch (Table 2). To verify whether the three types of designed sgRNAs functioned normally, rat fibroblasts were transfected with the Cas9 system and subjected to PCR-mediated T7E1 analysis. The first and second targets did not show T7E1 activity, similar to that of the negative control, but T7E1 activity was observed in the third target (Figure 2B). These results demonstrate that the CRISPR-Cas9-based fukutin gene depletion system was successfully constructed.

### 3.3. Production of Fukutin K/O Rat and Confirmation

**Figure 3:**
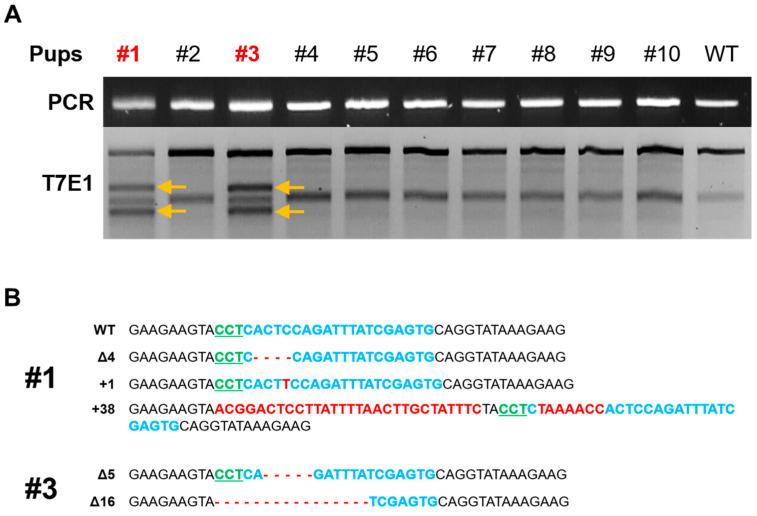
Production of fukutin gene-deficient rats and identification of target sequences. (**A**) T7E1 assay to identify fukutin-deficient rat litter. The arrow indicates that the gene targeting was successful. (**B**) Analysis of genetic defect types using DNA sequencing in the genomes of two founders 1 and 3. ∆ indicates deletion of DNA base and + indicates insertion of DNA base. Blue text indicates the target site, green text indicates the PAM sequence, and red text indicates the insertion of the DNA base.

**Figure 4:**
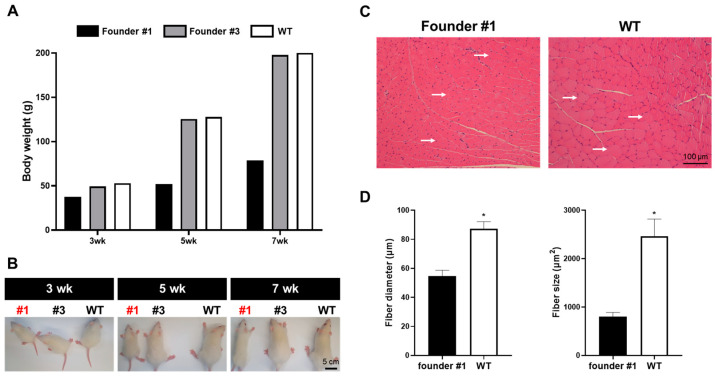
Comparison of morphology and skeletal muscle in founders. (**A**) The body weights of fukutin-deficient rats were compared at 3, 5, and 7 weeks, respectively. (**B**) Photograph of fukutin-deficient rats and WT at 3, 5 and 7 weeks. (**C**) Cross-sections of quadriceps muscles stained with H&E. (**D**) Analysis of fiber diameters and size in founder 1 and WT. Data are the means ± SE and analyzed by sStudent’s *t*-test. Values with different superscripts denote a significant difference compared with other groups (* *p* < 0.05). White arrows indicate fascicle diameter.

To produce fukutin KO mice, the previously identified sgRNA-c and Cas9 protein complexes were microinjected into the male zygote pronucleus, and 30 embryos per surrogate mother were transplanted into the oviducts. In total, 11 litters were born. Their tail tissues were extracted, and PCR and T7E1 assays were performed to confirm the targeting of the fukutin gene. T7E1 activity was confirmed in the two founders, and DNA sequencing was performed to verify the targeting of the fukutin gene (Figure 3A). Based on the DNA base sequences of the two founders, founder 1 was confirmed to have a deletion pattern of 4 bases and insertions of 1 and 38 bases, and founder 3 was confirmed to have deletions of 5 and 16 bases (Figure 3B). Additionally, for the phenotypic analysis, the size and body weight of the two fukutin KO founders were compared with those of wild-type pups born to the same surrogate mother at 3, 5, and 7 weeks of age. In the case of founder 1, the body weight was abnormally reduced to the same extent as in the wild type and founder 3 (Figure 4A,B). Additionally, skeletal muscle diameter and area decreased in founder 1 (Figure 4C,D). These results demonstrate that disease-specific fukutin gene KO transgenic rats were produced using the CRISPR/Cas9 system.

Regrettably, founder 1 rat passed away at 8 weeks of age. Subsequently, we conducted additional experiments to investigate the impact of fukutin deficiency on developmental timing in rats. Through comparisons of fetus size and genotype at embryonic day 13.5, achieved by crossing fukutin heterozygous rats, we confirmed that the size of fukutin gene double knockout fetuses was smaller compared to other genotypes (Figure S2).

## 4. Discussion

This is the first study to report the development of a fukutin KO rat model to study FCMD. Despite the relatively greater physiological similarities between humans and rats than between mice and humans [2], handling rats in the laboratory is more challenging than handling mice for the purpose of producing litter using transgenic embryos and establishing embryonic ES, which reduces the options available for establishing genetically modified animal models [3]. The CRISPR/Cas9 system is a useful tool for genome editing in various species. Its usage has increased notably over the past 20 years and is currently being applied to construct rat animal models [16,17]. We successfully used this efficient system to modify the rat genome and have developed the first rat model of MD.

Despite efforts to treat MD, early stage research in humans remains unsatisfactory because of the paucity of appropriate animal models and genomic studies to understand the pathogenesis of MD. However, a recent study using a mouse KO model reported that fukutin deficiency may contribute to MD pathogenesis [13]. Hence, MD research needs to be performed using various animal models, and the fukutin-deficient rat animal model established in this study may lead to advancements in MD research.

Obtaining a large number of fertilized eggs is the first and most crucial step in producing a transgenic rat model. A previous study reported that to obtain a large number of zygotes from Wistar–Imamichi rats for the optimal production of transgenic rats, female rats needed to be superovulated at the age of 10–15 weeks rather than at 6–9 weeks; moreover, hormones PMSG (150 IU/kg) and hCG (75 IU/kg) needed to administered [18]. Based on this information, we investigated the optimal superovulation conditions in SD rats for the current study, as SD rats are more sensitive to gonadotropins than are Wistar rats. As expected, our results confirm the differences in superovulatory conditions between Wistar and SD rats. Specifically, ovulation was higher when amounts of 150 IU/kg or more of both PMSG and hCG were administered than when the hormone concentration was 75 IU/kg. Notably, the high hormone concentration (250 IU/kg) group showed an increased number of embryo deaths and decline in embryo quality (Figure 1B). Additionally, when rat embryos were cultured in the M16 medium, which is normally used for mouse embryo development, the development rates at the two- to four-cell stage were 85.3%, 100%, and 81.5%, respectively, in each hormone concentration group. However, zygotes in all three groups failed to develop beyond the eight-cell stage, and embryonic development was halted (Table 1). Hence, the medium was replaced with the mR1ECM culture medium used by Miyoshi in 2016 [19]. This improved blastocyst development rates in all treatment group to 18.7%, 49.1%, and 45.2%, respectively, indicating unrestricted in vitro embryo development (Table 1). These results helped establish the optimal hormone injection concentration.

The CRISPR/Cas system is widely used as a powerful tool for the genetic modification of cells and animals. Many studies have used various tools to design CRISPR/Cas9 target sites [20,21,22]. Using the Rgen Tool program provided by the Institute for Basic Science Genome Editing Research Center, candidate groups were selected at two points in Exon 5 and 1 point in Exon 6. The target sequence recommended by the RGEN tool was the optimal target point with no off-target sequences among the 10 exons of fukutin (Figure 2A). However, because the efficiency of targeting had to be verified, a preliminary experiment was performed using T7E1 analysis, which enables the quick confirmation of gene targeting efficiency. We used three sgRNA candidates in rat fibroblasts [23,24] and confirmed the gene-targeting efficiency of the third candidate sgRNA; therefore, this candidate was used to produced function-deficient rats.

Several studies using various concentrations of sgRNA and Cas9 protein have been conducted since genome editing using recombinant Cas9 protein was first reported in rodent animal model embryos in 2015 [25]. Particularly, owing to the nature of the zygote, cell division occurs rapidly and continuously at the beginning. Therefore, compared with the method involving the injection of the Cas9 gene in the form of a plasmid, gene targeting is induced at a faster rate when Cas9 is injected as a protein. Therefore, we microinjected the selected sgRNA and synthesized Cas9 protein into rat zygotes, followed by embryo transfer. This method produced 11 litters. T7E1 analysis using the genome extracted from the tail tissue of the newborn rats showed that a transgenic rat population was established with approximately 20% efficiency. To confirm the exact target region of the two fukutin KO rats, TA cloning was performed using the PCR fragment. Since we microinjected the sgRNA–Cas9 complex into the pronucleus of the zygote before cleavage, we expected to observe two genotypes: wild-type and mutant. However, we confirmed four genotypes, as shown in Figure 3B. These results suggest that the injected sgRNA–Cas9 complex was active and continued to affect the diploid genome even during the of two-cell division stage. Additionally, significant phenotypic differences were observed between the two founders. At 3 weeks of age, founder 1 showed a reduced weight that was 70% of the body weight of the control group. At 7 weeks of age, the body weight reduced to 40% of that of the control group. This is consistent with the results obtained in the mouse model of MD [26]. However, in the case of founder 3, the body weight did not significantly differ compared with that of the control group, suggesting that the wild-type genome was acting dominantly in muscle-related cells. Unfortunately, founder 1 rat died at 8 weeks of age. We performed additional experiments to determine whether a deficiency of fukutin affects developmental timing in rats. We compared the size and genotype of the fetus implanted at embryonic day 13.5 by crossing fukutin heterogenous rats, and it was confirmed that the size of the fukutin gene double KO fetus was smaller than that of other genotypes (Figure S2). This fact is similar to the embryonic-lethal effect in fukutin-deficient mice reported by Kurahashi et al. in 2005 [27]. Hence, the generation of fukutin conditional knockout rats is deemed crucial for advancing research aimed at addressing muscular dystrophy.

## 5. Conclusions

In conclusion, we successfully used the CRISPR/Cas9 system to generate disease-specific transgenic rats exhibiting Fukuyama-type congenital muscular dystrophy. This rat model has extensive potential for application in research to determine the mechanisms underlying FCMD disease onset, symptoms, and treatment methods. However, further studies would need to be conducted before various physiological and pathological studies can be performed using this rat model.

## Figures and Tables

**Table 1:** Optimal hormone dose and IVC medium for embryonic development on rats.

PMSG/hCG (IU/kg)	2–4 Cell (%)		8–16 Cell (%)		Blastocyst (%)	
	M16	mR1ECM	M16	mR1ECM	M16	mR1ECM
75/75	85.0 ± 7.63	100.0 ± 0.00 *	0.0 ± 0.00	52.9 ± 18.06 *	0.0 ± 0.00	24.5 ± 19.25 *
150/150	100.0 ± 0.00 ^†^	98.8 ± 0.62	0.0 ± 0.00	78.3 ± 2.69 *^,†^	0.0 ± 0.00	49.0 ± 1.56 *^,†^
250/250	79.7 ± 9.86	93.4 ± 3.97 *	0.0 ± 0.00	66.8 ± 7.13 *	0.0 ± 0.00	45.1 ± 6.94 *

* Indicates significantly different by comparing M16 and mR1ECM medium (*p* > 0.05). ^†^ Indicate significantly different by comparing dose of PMSG/hCG (*p* > 0.05).

**Table 2:** Detail for target sgRNA.

Match Name	Coordinate	Spacer + PAM	Number of Mismatch	Out-of-Frame Score
*Fukutin* sgRNA-a	Chr 5: 73158279-73158257	GAGTCTATTCCATCCAGCCGAGG	0	77.5790797987
*Fukutin* sgRNA-b	Chr 5: 73158319-73158299	AGCTTACAGACATAGTGCAGAGG	0	73.0049498974
*Fukutin* sgRNA-c	Chr 5: 73160527-73160505	CACTCGATAAATCTGGAGTGAGG	0	84.1311264059

The results were obtained using Cas-OFFinder (http://www.rgenome.net/cas-offinder/; 1 May 2023).

## Data Availability

Data are contained within the article and Supplementary Materials.

## Supplementary Materials

The following supporting information can be downloaded at: https://www.mdpi.com/article/10.3390/cimb46050234/s1, Figure S1: Number of pups after embryo transfer. The zygotes were implanted into the oviduct four times in 30 embryos per surrogate mother rat. Figure S2: Fukutin hetero KO mice were crossed and embryo size and genotype were compared at embryonic day 13.5. It was confirmed that the size of the fukutin gene double KO fetus was smaller than that of other fetuses.
