# Supplementary material for: Optimizing Embryo Collection for Application of CRISPR/Cas9 System and Generation of Fukutin Knockout Rat Using This Method
Source: Curr Issues Mol Biol. 2024 Apr 23;46(5):3752–62. doi: 10.3390/cimb46050234 (PMC11120416; doi:10.3390/cimb46050234)
Supplement: Supplementary file 1 [file cimb-46-00234-s001.zip › cimb-2904380-supplementary.pdf]

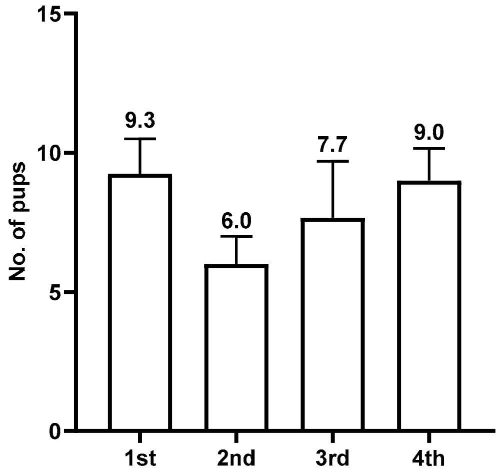

Figure S1. Number of pups after embryo transfer. The zygotes were implanted into the oviduct four times in 30 embryos per surrogate mother rat.

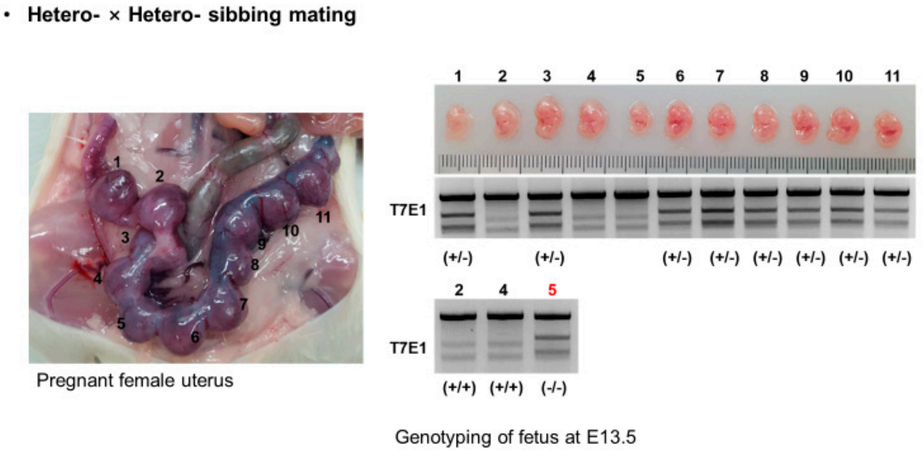

Figure S2. Fukutin hetero KO mice were crossed and embryo size and genotype were compared at embryonic day 13.5. It was confirmed that the size of the Fukutin gene Double KO fetus was smaller than that of other fetuses.
